# Crystallization and Electrical Properties of Ge-Rich GeSbTe Alloys

**DOI:** 10.3390/nano12040631

**Published:** 2022-02-14

**Authors:** Stefano Cecchi, Iñaki Lopez Garcia, Antonio M. Mio, Eugenio Zallo, Omar Abou El Kheir, Raffaella Calarco, Marco Bernasconi, Giuseppe Nicotra, Stefania M. S. Privitera

**Affiliations:** 1 Paul-Drude-Institut für Festkörperelektronik, Leibniz-Institut im Forschungsverbund Berlin e.V., Hausvogteiplatz 5–7, 10117 Berlin, Germany; eugenio.zallo@wsi.tum.de (E.Z.); raffaella.calarco@artov.imm.cnr.it (R.C.); 2 Department of Materials Science, University of Milano-Bicocca, via R. Cozzi 55, 20125 Milano, Italy; o.abouelkheir@campus.unimib.it (O.A.E.K.); marco.bernasconi@unimib.it (M.B.); 3 Institute for Microelectronic and Microsystems (IMM), National Research Council (CNR), Zona Industriale Ottava Strada 5, 95121 Catania, Italy; inakigarcia.lopez@imm.cnr.it (I.L.G.); antonio.mio@imm.cnr.it (A.M.M.); giuseppe.nicotra@imm.cnr.it (G.N.); stefania.privitera@imm.cnr.it (S.M.S.P.); 4 Walter Schottky Institut, Physik Department, Technische Universität München, Am Coulombwall 4, 85748 Garching, Germany; 5 Institute for Microelectronic and Microsystems (IMM), National Research Council (CNR), Via del Fosso del Cavaliere 100, 00133 Roma, Italy

**Keywords:** Ge-rich alloys, crystallization temperature, segregation, electrical properties

## Abstract

Enrichment of GeSbTe alloys with germanium has been proposed as a valid approach to increase the crystallization temperature and therefore to address high-temperature applications of non-volatile phase change memories, such as embedded or automotive applications. However, the tendency of Ge-rich GeSbTe alloys to decompose with the segregation of pure Ge still calls for investigations on the basic mechanisms leading to element diffusion and compositional variations. With the purpose of identifying some possible routes to limit the Ge segregation, in this study, we investigate Ge-rich Sb_2_Te_3_ and Ge-rich Ge_2_Sb_2_Te_5_ with low (<40 at %) or high (>40 at %) amounts of Ge. The formation of the crystalline phases has been followed as a function of annealing temperature by X-ray diffraction. The temperature dependence of electrical properties has been evaluated by in situ resistance measurements upon annealing up to 300 °C. The segregation and decomposition processes have been studied by scanning transmission electron microscopy (STEM) and discussed on the basis of density functional theory calculations. Among the studied compositions, Ge-rich Ge_2_Sb_2_Te_5_ is found to be less prone to decompose with Ge segregation.

## 1. Introduction

**Figure 1:**
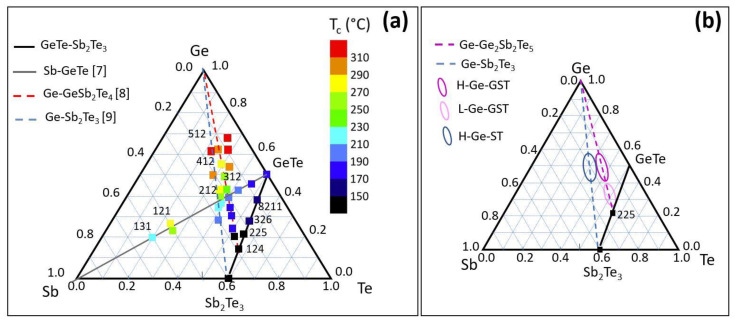
(**a**) Crystallization temperature of several compositions within the ternary GeSbTe phase diagram. The values have been taken from refs. [7,8,9]. (**b**) Compositions studied in this work.

GeSbTe (GST) chalcogenide alloys exhibit phase change properties that make this class of materials suitable for application in non-volatile memory technology [1,2,3,4]. In memory devices, the amorphous and crystalline phases are employed as logic states due to the large resistance contrast. The switching from the crystalline to the amorphous phases is obtained by applying a short, high-energy electrical pulse that melts the material, which is followed by a rapid thermal quench. A longer pulse at lower intensity is employed to recrystallize the memory cell. Such a phase change memory (PCM) technology is well assessed and already in production in the 20 nm node, with high potential to dominate the storage class memory sector, combining persistence and speed in the same device [5]. Despite the increasing technological impact, there are still some challenges that need to be solved, such as the low data retention, which presently can be a limiting factor for automotive or embedded applications, and the resistance drift, mainly affecting the amorphous phase. The data retention is limited by the crystallization temperature. The most commonly adopted materials for PCM have compositions belonging to the pseudo binary GeTe-Sb_2_Te_3_ tie-line of the GST ternary phase diagram, since no phase separation occurs after phase switching. Along this line, the crystallization temperature rises from 140 to 190 °C, as the GeTe amount increases [6]; therefore, it is not high enough to guarantee operation at high temperature, such as those required for embedded memories and for automotive applications. With the purpose of identifying materials with better data retention, without compromising the switching speed, GST alloys with composition off the GeTe-Sb_2_Te_3_ tie-line have been extensively studied in the literature, and the crystallization temperature for different alloys has been measured [7,8,9,10,11]. A survey of different GST compositions is reported in Figure 1. Among them, Ge-rich alloys have been proposed as valid candidates to address high-temperature applications due to their high crystallization temperatures. In the following, the Ge content in the alloys refers to the atomic percentage (e.g., for Ge_2_Sb_2_Te_5_ (GST225) at %(Ge) ≈ 22%).

In particular, Ge-rich Ge_2_Sb_1_Te_2_ was proposed as “golden composition”, since it showed a quite fast speed (about 80 ns) with a crystallization temperature of 250 °C [8]. Over the last years, several other compositions have been optimized in terms of cyclability, drift, and speed. However, the high crystallization temperature of Ge-rich alloys seems to be due to the slowing down of the crystallization kinetics produced by the mass transport involved in the phase separation process into crystalline Ge and GST alloys less rich in Ge [12,13]. Such a tendency to decompose still requires investigation on the basic mechanisms leading to compositional variations, since these may produce retention degradation upon cycling and even device failure. Recently, atomistic simulations applied to amorphous Ge-rich GST along the Ge-Ge_1_Sb_2_Te_4_ line have revealed that these alloys tend to separate into Ge and Ge_1_Sb_2_Te_4_ even in the amorphous phase when the Ge content is above 50% [14]. Thus, such a phase separation process is more likely to occur upon crystallization. The phase separation of Ge-Ge_1_Sb_2_Te_4_ alloys during crystallization has been very recently studied by means of high-throughput density functional theory (DFT) calculations [15]. It has been shown that the Ge-rich Ge_1_Sb_2_Te_4_ alloys become more prone to decompose into crystalline Ge and a GST alloy less rich in Ge, as Ge amount is above 50 at %. Therefore, it is crucial to understand to which extent the excess Ge segregates in Ge-rich GST alloys since, once impoverished in Ge, the remaining material may revert to stoichiometric phases that crystallize at lower temperatures. Moreover, the effect of the annealing thermal history on crystallization and segregation in Ge-rich GST has been shown by Di Biagio et al. [16]. The optimization of the microstructure of the crystallized films may influence also the switching properties.

In order to assess the relationship between amorphous stability and alloy composition in terms of structure and electrical properties, we investigate Ge-rich alloys with composition in the regions of the ternary phase diagram shown in Figure 1b. The electrical resistance as well as the crystallization process and the formed phases are investigated as a function of annealing temperature. The experimental observations have been corroborated by DFT calculations, which allow for the identification of the possible decomposition pathways during crystallization and therefore suggest some viable routes to limit the Ge segregation.

## 2. Materials and Methods

Amorphous chalcogenide Ge-rich GST films were deposited either on Si(111)-(√3 × √3)R30°-Sb passivated surfaces or on SiO_2_ substrates. The samples were fabricated by molecular beam epitaxy (MBE), and the substrate temperature was kept at room temperature. The film thickness was 30–60 nm, and the growth rate was ≈0.3–0.6 nm/min depending on film composition. As shown in Figure 1b, the studied compositions are Ge-rich Sb_2_Te_3_ with a high amount of Ge (>40 at %), which will be indicated in the following as H-Ge-ST; Ge-rich GST225, with a low (<40 at %) or high amount of Ge, which will be indicated in the following as L-Ge-GST and H-Ge-GST, respectively. For Ge-rich GST225 samples, the temperature of the Ge effusion cell was correspondingly set to achieve the two compositions, while all the other growth parameters were unchanged.

The films grown on Si(111) were capped in situ by a sputtered ZnS:SiO_2_ layer and annealed ex situ by means of rapid thermal annealing (RTA). The annealing treatments were performed at increasing temperatures in the range of 140–330 °C for 30 min each under 1 bar nitrogen atmosphere. The RTA ramp-up time was set to 10 s for all temperatures. After each annealing step, the structural properties have been followed ex situ by X-ray diffraction (XRD) in order to study the crystallization and segregation processes.

For XRD measurements, a four-circle PANalytical X’Pert Pro Material Research Diffractometer system (Malvern Panalytical Ltd., Malvern, UK), equipped with a Ge (220) hybrid monochromator and Cu Kα1 X-ray radiation (λ = 1.540598 Å), has been employed. The curves were acquired in ω-2θ configuration along the symmetric rod of the Si(111) substrate.

The electrical properties have been investigated by in situ sheet resistance measurements of the samples grown on SiO_2_ with a four-point probe, during annealing in air using a Temptronic ThermoChuck system (inTEST Thermal Solutions GmbH, Müllrose, Germany). The temperature dependence of the resistance has been evaluated in the range from 30 to 300 °C by employing a Agilent HP 4156B parameter analyser (Agilent Technologies, Inc., Santa Clara, CA, USA).

The crystalline structure and the element distribution after crystallization have been investigated by scanning transmission electron microscopy (STEM) and electron energy loss spectroscopy (EELS). The microscopical analyses have been performed by using a JEOL ARM200F (JEOL Ltd., Tokyo, Japan) Cs-corrected microscope, which is equipped with a cold-field emission gun and operating at 200 keV. Micrographs were acquired in Z-contrast mode by high-angle annular dark field (HAADF). A GIF Quantum ER system (Gatan AMETEK, Pleasanton, CA, USA) was used for EELS measurements. Both low- and core-loss EELS spectra were acquired with the Dual EELS tool through Gatan DigitalMicrograph software Version 3.4 (Gatan AMETEK, Pleasanton, CA, USA), in spectrum imaging (SI) mode.

Finally, we have computed the reaction free energy for the decomposition pathways of H-Ge-ST and H-Ge-GST alloys, by using the DFT data on the formation free energy of cubic alloys in the central part of the ternary Ge-Sb-Te phase diagram reported in our previous work, [15] which we refer to for all details.

## 3. Results and Discussion

**Figure 2:**
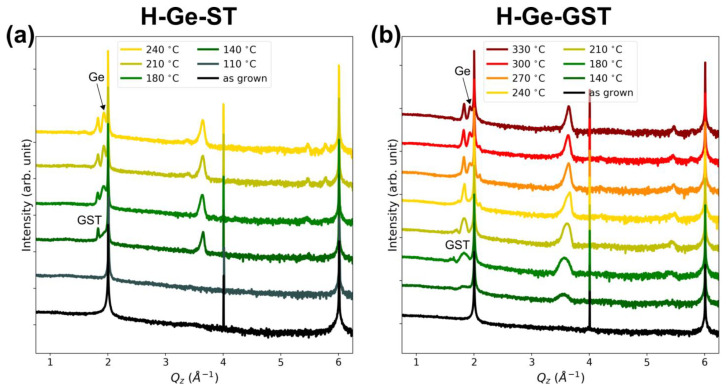
XRD ω-2θ scans as a function of annealing temperature for (**a**) H-Ge-ST (Ge-rich Sb_2_Te_3_), and (**b**) H-Ge-GST (Ge-rich Ge_2_Sb_2_Te_5_).

Figure 2a,b shows the XRD ω-2θ scans acquired after different annealing steps at increasing temperature for H-Ge-ST and H-Ge-GST, respectively. The sharp peaks at ≈2, 4, and 6 Å^−1^ correspond to the 111, 222, and 333 Bragg reflections of the Si substrate. In both alloys, a GST phase with rocksalt structure is formed at low annealing temperatures (see peaks at ≈1.83, 3.66, 5.49 Å^−1^) [17,18], whilst higher temperatures are required to observe the crystallization of segregated germanium (peaks at ≈1.93 and 5.79 Å^−1^ for 111 and 333 Bragg reflections, respectively). Although the two alloys have equal Ge content, the temperature at which crystallization occurs is different. In particular, in H-Ge-ST, the onset of crystallization of the GST rocksalt phase occurs at 140 °C, and the segregated Ge crystallizes at about 180 °C. For H-Ge-GST, crystallization shifts to higher temperatures, with the formation of rocksalt GST between 180 and 210 °C (crystallization onset still at 140 °C) and the Ge crystallization at 270 °C, respectively. In addition, we notice the intensity ratio between GST and Ge peaks in the two samples after crystallization, which qualitatively suggests their different tendency to decompose. The weak peak at ≈3.38 Å^−1^ that appears for H-Ge-ST at 210 °C may be attributed to segregated Sb, as confirmed by STEM and EELS analysis (see afterwards). Therefore, these data already suggest the higher stability of H-Ge-GST with respect to H-Ge-ST.

XRD data of sample L-Ge-GST, upon annealing up to 300°C, indicate the presence of a single GST phase and no Ge segregation (see Figure S1 in the Supplementary Materials).

**Figure 3:**
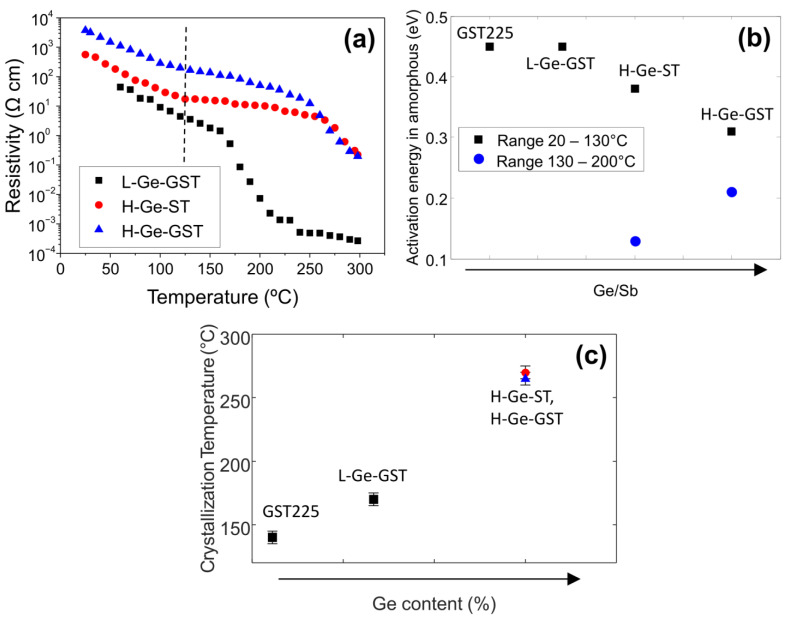
(**a**) Resistivity as a function of temperature for the studied compositions. (**b**) Activation energy for conductivity in the amorphous phase as a function of the ratio between Ge and Sb. (**c**) Crystallization temperature as obtained from the first derivative of the resistivity versus temperature as a function of Ge content.

Figure 3a shows the resistivity versus annealing temperature as measured in situ after 3 min isothermal annealing for all the studied compositions. The resistivity of the as-grown amorphous phase increases as the Ge content increases, and the highest value is measured for H-Ge-GST. For the composition with lower Ge amount (L-Ge-GST), the temperature dependence of the resistivity of the amorphous phase corresponds to an activation energy of 0.45 eV, which is a value typical of amorphous Ge_2_Sb_2_Te_5_ (GST225). At about 160 °C, the resistivity sharply decreases due to the film crystallization. Hence, consistently with previous studies, the crystallization temperature is higher with respect to a reference GST225 [9]. The resistance variation from amorphous to crystalline phase for L-Ge-GST is about four orders of magnitude.

For higher Ge content, the activation energy for the conductivity of the amorphous phase is slightly lower than that for GST225 and decreases as the Ge/Sb ratio decreases, as shown by black symbols in Figure 3b. Interestingly, at about 130 °C, the dependence of the resistivity versus temperature further reduces (see dashed line) and, as a consequence, the resistivity remains above 1 Ω cm up to 230 °C. The activation energies for amorphous conductivity obtained in the range 130–200 °C are shown in Figure 3b as blue symbols. In this case, the activation energy increases with the Ge/Sb ratio. These observations indicate that (i) up to 230 °C, the electrical conduction properties of H-Ge-GST and H-Ge-ST are mainly determined by the conductivity of the amorphous matrix; (ii) still, modifications of its composition are occurring, contextually to the nucleation of the GST crystallites, and such a process is more noticeable in H-Ge-ST. Finally, from the first derivative of the resistivity versus temperature, the effective crystallization temperature of the films has been evaluated as the temperature at which the derivative exhibits a minimum. It is worth noting that in the case of alloys in which different phases are formed upon annealing, as observed by XRD for H-Ge-GST and H-Ge-ST samples, the crystallization temperature obtained from the electrical measurements does not correspond to the crystallization of one single phase. Instead, it represents an effective value corresponding to the temperature at which a percolation path between regions with low resistance may be established. The results reported in Figure 3c clearly indicate that the effective crystallization temperature is dominated by the Ge amount. The two alloys with equal high Ge content, H-Ge-ST and H-Ge-GST, exhibit the same effective crystallization temperature within errors.

In the case of the alloys with higher Ge content, we also observe that the resistance contrast between the amorphous and the crystalline phases is about two orders of magnitude (see Figure 3a). This value, lower than that of GST225 and L-Ge-GST, is still enough to ensure distinguishing between two logic states [19]. However, we note that in these samples, the saturation of the low resistance value is not reached up to 300 °C. Due to limitations of the employed experimental apparatus, in situ annealing at temperatures above 300 °C could not be performed. In addition, TEM analysis of the sample H-Ge-ST annealed at 300 °C (see Figure S2 in the Supplementary Materials) shows that the film is still partially amorphous. Therefore, upon complete crystallization, the resistance contrast is expected to be larger than two orders of magnitude.

**Figure 4:**
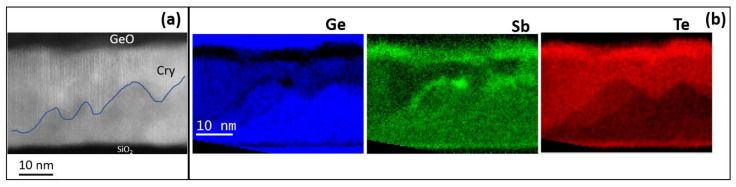
(**a**) STEM micrograph of the film H-Ge-ST after annealing in air at 180 °C. The crystallized (Cry) region is indicated in the figure. (**b**) Ge, Sb, and Te elemental maps obtained by EELS spectra acquired in SI mode. The intensity of the signal corresponding to the regions of Ge, Sb, and Te in the EELS spectra is reported in false colors.

In order to get more insight in particular on the microstructure of the crystallized films, we have performed STEM and EELS analyses of the two alloys with higher Ge content. Figure 4a shows an STEM micrograph of the H-Ge-ST film on SiO_2_ (uncapped) after annealing in the same setup used for electrical measurements at 180 °C for 30 min. The sample has been prepared aiming to investigate the modifications of the amorphous phase composition occurring at temperatures in the range 130–230 °C, as suggested by the electrical characterization. The upper part of the film shows several crystalline regions, whilst the bottom part is still mainly amorphous. The formation of a thin GeO layer at the film surface is also observed, suggesting that Ge oxidation may facilitate the crystallization of the underlying film, since it remains less rich in Ge. Figure 4b shows the elemental maps obtained by EELS spectra acquired in each pixel in SI mode. The elemental analysis shows the GeO topmost layer, appearing bright in the Ge map. In the same Ge map, the dark layer below the oxide is a crystalline region in which Ge is heavily depleted and Sb and Te correspondently exhibit higher concentrations. The crystalline regions, which form below the Ge-depleted region, contain less Ge atoms. On the contrary, higher Ge concentration is observed in the residual amorphous regions at the bottom of the film. This indicates that Ge atoms move (i) toward the surface, where they are oxidized, leaving regions depleted of Ge below the surface oxide; and (ii) toward the bottom of the film, enriching the amorphous fraction. Therefore, in the uncapped film, the Ge-depleted region may crystallize at lower temperature, favoring the further crystallization of GST. The reduction of the crystallization temperature in uncapped Ge_2_Sb_2_Te_5_ films exposed to air, due to the selective oxidation of Ge, and to a minor extent of Sb, has been in fact extensively reported in the literature [20,21,22,23,24,25,26]. This behavior has been also recently confirmed in Ge-rich GST [27].

This experiment provided evidence of the formation of less Ge-rich crystalline grains, in this case heterogeneously nucleating underneath the Ge-depleted region, which is compatible with the crystallization observed in the XRD data at temperatures below 180 °C. Despite such evolution of the film microstructure, the results of the electrical measurements indicate that the electrical resistivity of the film remains very high up to 270 °C, while we record a change in the activation energy for the conductivity of the amorphous phase. This is because the crystalline grains, having lower Ge concentration, in order to grow expel the excess Ge, which therefore accumulates in the residual amorphous regions. Therefore, the amorphous fraction is expected to become richer and richer in Ge as the annealing temperature increases and the less-Ge rich crystalline grains grow. Still, as far as the crystalline grains are embedded into an amorphous matrix, and no percolation path is available, the electrical properties of the material are dominated by the part of the film which is still not crystallized. Ultimately, the change of activation energy highlighted in Figure 3a can be explained by considering the Ge enrichment of the amorphous matrix. Further details can be found in the Supplementary Materials: Figures S3 and S4.

**Figure 5:**
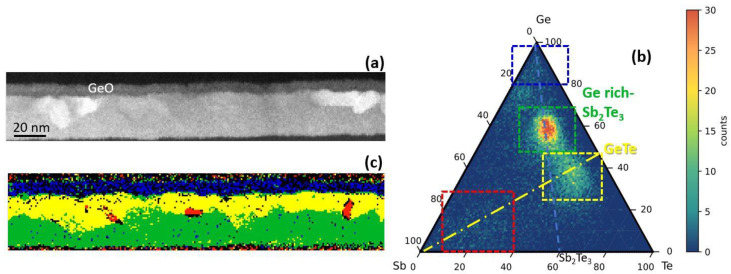
(**a**) STEM image of H-Ge-ST film on SiO_2_ after annealing at 300 °C. (**b**) Ternary GeSbTe diagram built by merging the atomic maps distribution obtained by EELS spectra acquired in SI mode. (**c**) Stoichiometric map extracted from the ternary diagram. Ge-rich, Sb-rich, GeTe-rich, and Ge-rich Sb_2_Te_3_ regions are marked in blue, red, yellow, and green, respectively.

Figure 5a shows an STEM micrograph of the same H-Ge-ST uncapped film on SiO_2_ after annealing up to 300 °C for 30 min. The bottom part of the film is still partially amorphous (see also Figure S2). The higher contrast of some grains in Figure 5a is due to their alignment to the zone axis. By merging the elemental maps obtained by EELS, we extracted a ternary GST phase diagram, as shown in Figure 5b, and the corresponding stoichiometric map (Figure 5c). In the stoichiometric map of Figure 5c, regions with composition within a given range are plotted with the same color. The compositional ranges are shown in Figure 5b (dashed rectangles). The crystalline grains observed below the surface oxide have mainly a composition close to GeTe, with some Sb amount (yellow regions), whilst the bottom part of the film has a Ge-rich Sb_2_Te_3_ composition, as shown in green, with a higher Ge amount than that in the as-grown sample. Some regions, reported in red, have an Sb rich composition, which indicates that Sb tends to accumulate at the grain boundaries or in defective regions.

**Figure 6:**
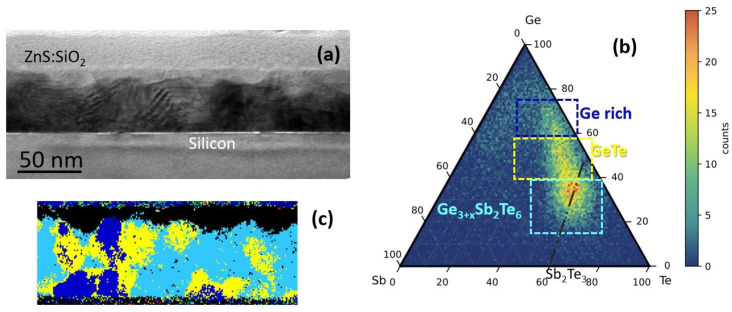
(**a**) STEM micrograph of H-Ge-GST on Si(111) after annealing at 330 °C. (**b**) Ternary GeSbTe diagram built by merging the atomic maps distribution obtained by EELS spectra acquired in SI mode. (**c**) Stoichiometric map extracted from the ternary diagram. Ge-rich, GeTe-rich, and Ge_3+x_Sb_2_Te_6_ regions are marked in blue, yellow, and light blue, respectively.

Figure 6a shows the STEM micrograph of the H-Ge-GST film deposited on Si(111) and annealed up to 330 °C for XRD characterization. For this sample, we recall that a ZnS:SiO_2_ layer was deposited by in situ sputtering to prevent the oxidation during the annealing study. First, it is relevant to evaluate the overall crystallization status of the film. After the seven annealing steps from 140 to 330 °C, the material is completely crystalline, confirming our interpretation of the electrical measurements. Then, the ternary GST phase diagram extracted from the EELS elemental maps is reported in Figure 6b, and the distribution of the phases is shown in Figure 6c. The largest part of the film crystallizes preferentially with a composition of Ge_3+x_Sb_2_Te_6_ (shown in light blue), which is close to the GeTe-Sb_2_Te_3_ pseudobinary line (reported in Figure 6b as black dashed line). Ge-rich regions (in blue) and with stoichiometry close to GeTe (in yellow) are also observed.

Further TEM analyses, including selected area electron diffraction, are reported in Figures S5 and S6 in the Supplementary Materials.

The experimental findings can be better understood with the support of DFT calculations. In a previous work [15], we have computed the DFT formation free energy, with respect to the elements in their standard states, of cubic GST alloys in the central region of the ternary phase diagram. The free energy consisted of the total energy at zero temperature and the configurational free energy (at room temperature) due to disorder in the cubic sublattices. The calculated formation free energy is lower for alloys along the GeTe-Sb_2_Te_3_ tie-line and on the Sb-GeTe isoelectronic line [15].

This information can be used to compute the reaction free energy of the decomposition of a given cubic alloy with composition Ge_x_Sb_y_Te_z_ along all possible pathways given by

Ge_x_Sb_y_Te_z_ → a Ge_h_Sb_k_Te_m_ + b Sb + c Ge + d Te + e Sb_2_Te_3_ + f GeTe (1)

Calculations on selected cases have shown that the vibrational contribution to the reaction free energy is negligible [28].

For each alloy, we can then construct a map of decomposition free energy, which highlights the more probable decomposition paths occurring during crystallization from the amorphous phase. In Ref. [15], this scheme was exemplified by studying the decomposition pathways of Ge-rich alloys on the Ge-GeSb_2_Te_4_ tie-line. Following the same methodology, we have computed the decomposition maps for the two alloys with high Ge amounts studied here.

**Figure 7:**
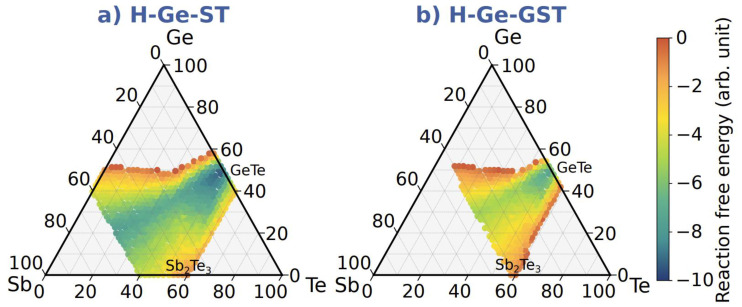
Color map of decomposition pathways during crystallization for (**a**) H-Ge-ST and (**b**) H-Ge-GST obtained from DFT data on the formation free energies in Ref. [15].

Figure 7 shows the map of decomposition pathways during crystallization for H-Ge-ST (a) and H-Ge-GST (b). The decomposition paths have been calculated for the nominal stoichiometries of H-Ge-ST and H-Ge-GST samples. Each colored point for the generic alloy Ge_h_Sb_k_Te_m_ gives the value of the decomposition free energy for the formation of Ge_h_Sb_k_Te_m._ Negative reaction free energy indicates an exothermic reaction, while the green and blue regions correspond to the more favored decomposition pathways.

Only exothermic reactions forming Ge_h_Sb_k_Te_m_ products that correspond at least to 33 at % in reaction (1) are reported in the map. Only under these conditions do we consider an alloy the main product of the decomposition pathway and show a point on the decomposition map.

We note that for H-Ge-ST, the reaction free energy is negative for a wider part of the phase diagram, indicating that several compositions may be formed. The most exothermic reactions lead to the formation of alloys on the Sb-GeTe and GeTe-Sb_2_Te_3_ lines, with a larger weight for alloys close to GeTe. Thus, we may expect the existence of different cubic alloys as the results of crystallization of amorphous Ge-rich Sb_2_Te_3_. In comparison, far fewer exothermic pathways are obtained for the decomposition of H-Ge-GST, suggesting that this alloy should have a lower tendency to decompose.

**Figure 8:**
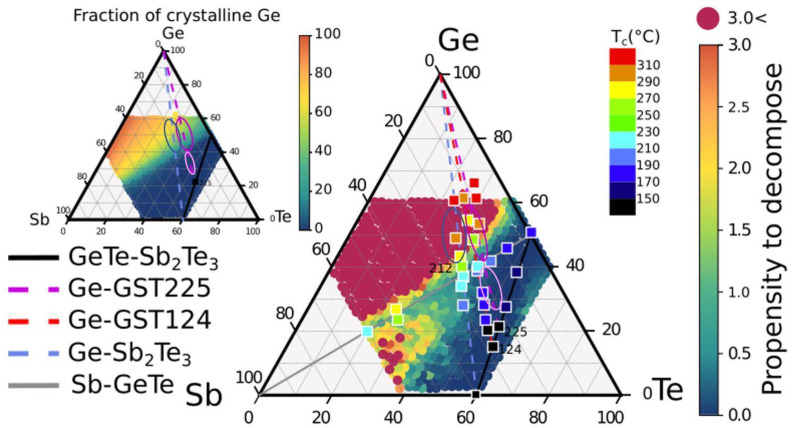
Crystallization temperature of several alloys studied in literature. Experimental data are plotted on the map obtained in Ref. [15] by DFT calculations showing the propensity to decompose (in eV/atom, see text). The inset on the left upper part shows the average crystalline Ge fraction upon crystallization, as obtained from DFT calculations in Ref. [15]. The compositions studied in this work, as already shown in Figure 1b, are indicated by ellipses.

In our previous work [15], we introduced a measure of the decomposition propensity to quantify how much a given alloy is prone to decompose. This is obtained by counting the exothermic decomposition channels, weighted for their reaction free energy, including now all the possible products, even if present only in a small atomic fraction. The resulting map shows that the decomposition propensity is low along the GeTe-Sb_2_Te_3_ tie-line and also along the Sb-GeTe isoelectronic line [15]. In order to compare the theoretical results with the experimental data, and to evaluate possible strategies to tune the composition and obtain high crystallization temperature while minimizing the decomposition propensity, in Figure 8, we report as full squares the crystallization temperature of several compounds, as given in Figure 1. Circular symbols represent the calculated decomposition propensity. Blue points correspond to compositions with low propensity to decompose. L-Ge-GST (violet ellipse), that we found to exhibit no phase separation upon crystallization (see Figure S1), belongs to this group, together with other compositions that are characterized by crystallization temperature lower than 220 °C. Red circular points correspond to compositions with a high propensity to decompose. Alloys with higher crystallization temperature (>270 °C) are found within the red region, and therefore, they are expected to undergo massive phase separation upon crystallization. The composition H-Ge-ST (blue ellipse) is within a region with high propensity to decompose. The average fraction of Ge segregation for all alloys in the central part of the GeSbTe phase diagram was also computed in Ref. [15]. This map is shown in the inset on the left of Figure 8, which further highlights that a lower fraction of Ge segregates in the crystallization process of alloys on the Ge-Ge_2_Sb_2_Te_5_ tie-line than for alloys on the Ge-Sb_2_Te_3_ line. It is worth noting that the high crystallization temperature of Ge-rich alloys has been ascribed to the phase separation process which, requiring the diffusion of the atomic species on a long length scale, slows down the crystallization kinetics [13]. However, such an explanation clearly limits the possibility to select an alloy with sufficiently high crystallization temperature that is able to homogeneously switch with no phase separation, which is desired to minimize the cell-to-cell variation and the resistance drift of the crystalline state but is jeopardized by the presence of nanocrystals with different compositions or by residual amorphous regions. To this end, a trade-off is represented by compositions lying at the edges between regions with high and low propensity to decompose. Among these, we find the composition Ge-rich Ge_2_Sb_1_Te_2_ with Ge below 50 at %, which has been already identified in the past [8], and the H-Ge-GST (magenta ellipse) studied in this work. Both compositions are expected to segregate no more than 30% of the total amount of Ge. Nevertheless, the crystallization temperature is about 270 °C. Such a result suggests that the mass transport involved in the phase segregation may play a role in raising the crystallization temperature, but it cannot be the unique mechanism. Other factors should be taken into account, such as, for example, the dependence of the structural properties of the amorphous phase on composition and thermal history [16]. Indeed, it has been shown by previous analysis of DFT models that by increasing Ge [14,28] or Sb [29] content in respect to the GeTe-Sb_2_Te_3_ tie-line, the amorphous network becomes more dissimilar from the cubic crystal. This is because of the formation of more Ge-Ge bonds with tetrahedral geometry, at the expense of octahedral bonding, which characterizes the rocksalt phase. Moreover, according to molecular dynamics simulations of the crystallization process [4,30,31,32], the key process in the formation of cubic crystalline nuclei is the reorientation of the four-membered rings. By increasing the fraction of Ge and Sb, the number of four-membered rings decreases at the expense of longer rings. The presence of a larger amount of homopolar bonds and tetrahedra, promoted by the excess Ge, and the concomitant decrease in the number of four-membered rings might slow down the crystallization kinetics either by reducing the nucleation rate or the crystal growth velocity, which overall leads to an increase in the crystallization temperature. Further increase in the crystallization temperature without increasing the Ge segregation may also be achieved by different approaches, such as nitrogen or carbon doping.

## 4. Conclusions

In this paper, we have studied the stability and the electrical properties of Ge-rich GeSbTe alloys belonging to the Ge-Sb_2_Te_3_ and Ge-Ge_2_Sb_2_Te_5_ lines. The Ge-rich GST225 film with Ge amount lower than 40 at % has a crystallization temperature of 180 °C and exhibits no phase separation. Both Ge-Sb_2_Te_3_ and Ge-Ge_2_Sb_2_Te_5_ alloys with higher Ge amount (>40%) are characterized by the same higher crystallization temperature (270 °C) and by phase separation. However, depending on the composition, a different propensity to decompose is observed experimentally and evaluated by DFT calculations, with a lower fraction of segregated Ge for the composition H-Ge-GST. Therefore, this alloy is expected to have a better stability upon cycling in a phase change memory.

## Data Availability

The data that support the findings of this study are available from the corresponding author upon reasonable request.

## Supplementary Materials

The following are available online at https://www.mdpi.com/article/10.3390/nano12040631/s1, Figure S1: XRD ω-2θ scans obtained after annealing at 300 °C for H-Ge-GST (solid line) and L-Ge-GST (dotted line), Figure S2: High-resolution TEM image of H-Ge-ST after annealing at 300 °C for 30 min, Figure S3: Calculated resistivity of GST225 and Sb_2_Te_3_ according to the effective medium approximation, assuming the presence of some amount of excess Ge, Figure S4: Calculated resistivity of Sb_2_Te_3_ as a function of temperature, according to the effective medium approximation, assuming the presence of different amounts of excess Ge, Figure S5: (a) Bright field TEM and (b) SAED of the sample H-Ge-ST on SiO_2_ after annealing in air at 180 °C. The SAED is obtained from a selected area of about 100 nm of diameter, Figure S6: (a) Bright field TEM and (b) SAED of the sample H-Ge-GST on Si(111) after annealing at 330 °C. The SAED is obtained from a selected area of about 100 nm of diameter. Reflections marked in yellow correspond to the Si substrate along the [110] zone axis. The other reflections (in red) are compatible with Sb_2_Te_3_ and trigonal (GeTe)_n_(Sb_2_Te_3_).
