# Supplementary material for: Crystallization and Electrical Properties of Ge-Rich GeSbTe Alloys
Source: Nanomaterials (Basel). 2022 Feb 14;12(4):631. doi: 10.3390/nano12040631 (PMC8876497; doi:10.3390/nano12040631)
Supplement: Supplementary file 1 [file nanomaterials-12-00631-s001.zip › nanomaterials-1554934-supplementary.pdf]

nanomaterials

MDPI

Supplementary Materials

Crystallization and Electrical Properties of Ge-Rich GeSbTe Alloys

Stefano Cecchi [1,2,*], Iñaki Lopez Garcia [3], Antonio M. Mio [3], Eugenio Zallo [1,4], Omar Abou El Kheir [2], Raffaella Calarco [1,5], Marco Bernasconi [2], Giuseppe Nicotra [3] and Stefania M. S. Privitera [3]

1 Paul-Drude-Institut für Festkörperelektronik, Leibniz-Institut im Forschungsverbund Berlin e.V., Hausvogteiplatz 5–7, 10117 Berlin, Germany; eugenio.zallo@wsi.tum.de (E.Z.); raffaella.calarco@artov.imm.cnr.it (R.C.)
2 Department of Materials Science, University of Milano-Bicocca, via R. Cozzi 55, 20125 Milano, Italy; o.abouelkheir@campus.unimib.it (O.A.E.K.); marco.bernasconi@unimib.it (M.B.)
3 Institute for Microelectronic and Microsystems (IMM), National Research Council (CNR), Zona Industriale Ottava Strada 5, 95121 Catania, Italy; inakigarcia.lopez@imm.cnr.it (I.L.G.); antonio.mio@imm.cnr.it (A.M.M.); giuseppe.nicotra@imm.cnr.it (G.N.); stefania.privitera@imm.cnr.it (S.M.S.P.)
4 Walter Schottky Institut and Physik Department, Technische Universität München, Am Coulombwall 4, 85748 Garching, Germany
5 Institute for Microelectronic and Microsystems (IMM), National Research Council (CNR), Via del Fosso del Cavaliere 100, 00133 Roma, Italy
* Correspondence: stefano.cecchi@unimib.it

S1. Segregation of Ge in Ge-GST samples

X-ray diffraction (XRD) data obtained after annealing at 300 °C for H-Ge-GST and L-Ge-GST are compared in Figure S1. A single crystalline phase is clearly observed in the L-Ge-GST sample, without Ge segregation. On the contrary, the H-Ge-GST exhibits diffraction peaks ascribed to crystalline Ge (see main text page 4).

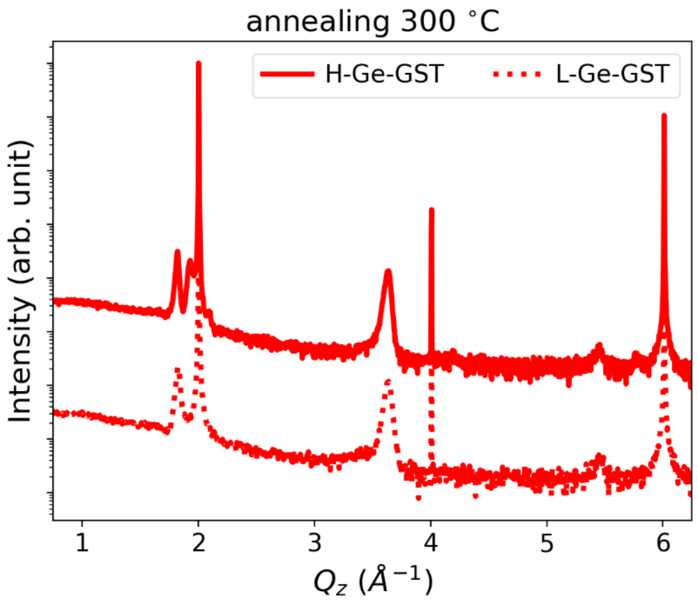

Figure S1. XRD ω-2θ scans obtained after annealing at 300 °C for H-Ge-GST (solid line) and L-Ge-GST (dotted line).

S2. Morphology of H-Ge-ST after annealing at 300 °C

The high-resolution transmission electron microscopy (TEM) image of H-Ge-ST, after annealing at 300 °C for 30 min, is shown in Figure S2. The film is uncapped and a thin

oxide layer is formed at the surface. Crystal nucleation preferentially occurs below the surface oxide, while the regions at the bottom of the film are still in the amorphous phase.

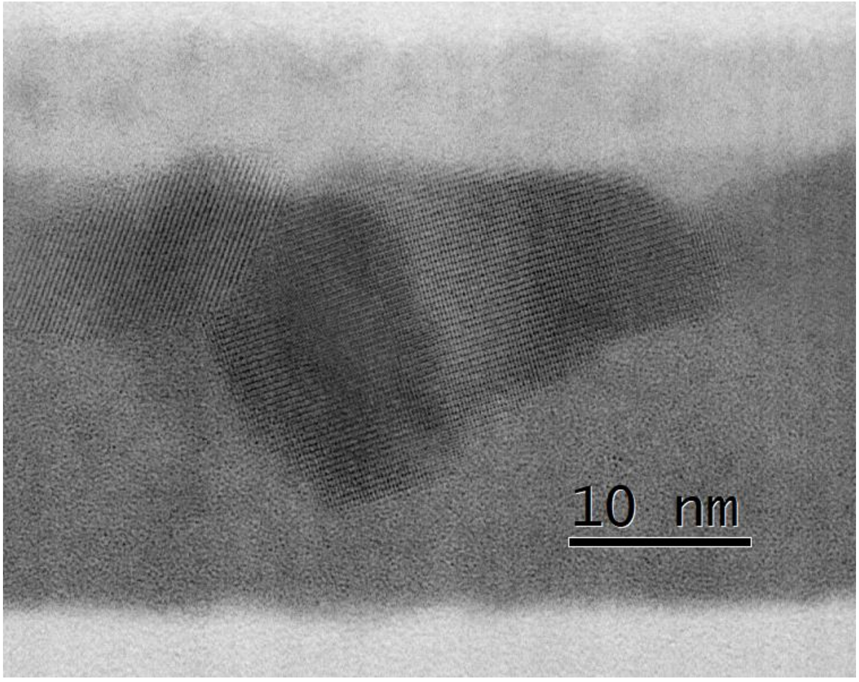

Figure S2. High-resolution TEM image of H-Ge-ST after annealing at 300 °C for 30 min.

S3. Effect of Ge enrichment of the amorphous phase

The electrical resistivity of inhomogeneous materials, as Ge-rich GST or ST films, can be evaluated by considering the effective medium approximation developed by Bruggeman [1]. Ref. [2] describes how to calculate the effective conductivity for various shapes of composite materials as a function of the "dopant" and the host material properties. In this case the host is GST225 or Sb_2Te_3 and the "dopant" is the excess Ge. Figure S3 shows the resistivity of Ge rich amorphous alloys as a function of excess Ge calculated according to Ref. [2].

For excess Ge amount below the percolation threshold (67%), the resistivity is expected to increase as the Ge content increases. Above the threshold, the material becomes more similar to amorphous Ge.

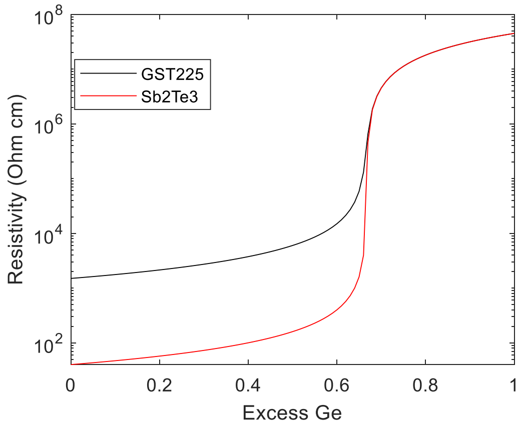

Figure S3. Calculated resistivity of GST225 and Sb_2Te_3 according to the effective medium approximation, assuming the presence of some amount of excess Ge.

Following the same approach, it is also possible to calculate the expected temperature dependence of resistivity as a function of excess Ge. Figure S4 shows the calculated temperature dependence of the resistance for different excess Ge amounts in Sb_2Te_3. As the Ge content in the amorphous phase increases, the activation energy for the resistivity decreases.

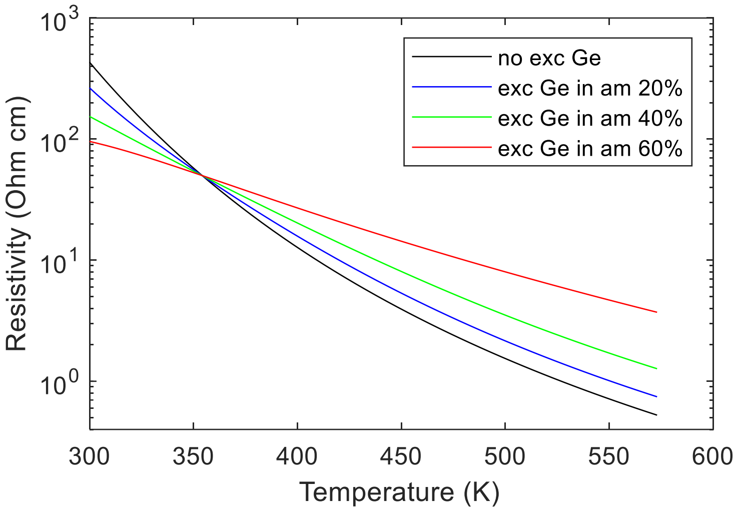

Figure S4. Calculated resistivity of Sb_2Te_3 as a function of temperature, according to the effective medium approximation, assuming the presence of different amounts of excess Ge.

S4. Selective area electron diffraction

Selected area electron diffraction (SAED) was performed on the samples H-Ge-ST and H-Ge-GST. The bright field TEM and SAED of sample H-Ge-ST on SiO_2 after annealing in air at 180 °C are reported in Figure S5. The plane spacings in the SAED are compatible with GeTe for a grain along the zone axis [1$\bar{1}$2].

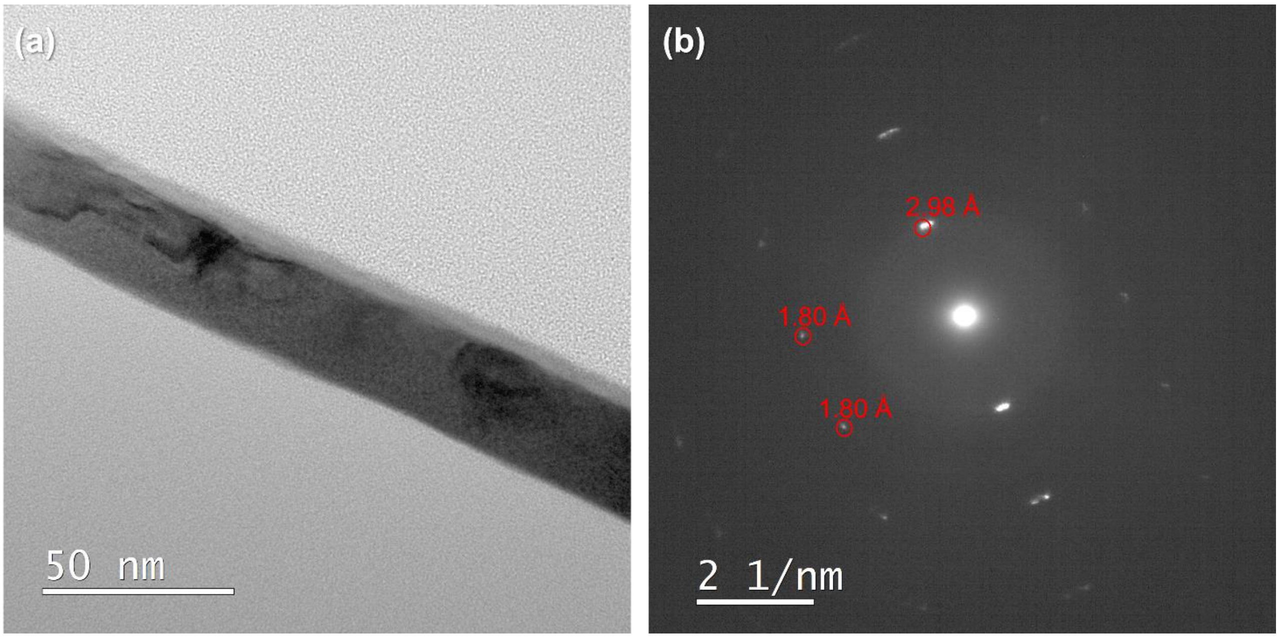

Figure S5. (**a**) Bright field TEM and (**b**) SAED of the sample H-Ge-ST on SiO_2 after annealing in air at 180 °C. The SAED is obtained from a selected area of about 100 nm of diameter.

In Figure S6 the bright field TEM and SAED of the sample H-Ge-GST on Si(111) after annealing at 330 °C are shown. Along with Si reflections (yellow circles), features compatible with Sb_2Te_3 and trigonal $(GeTe)_n(Sb_2Te_3)$ are seen (red circles).

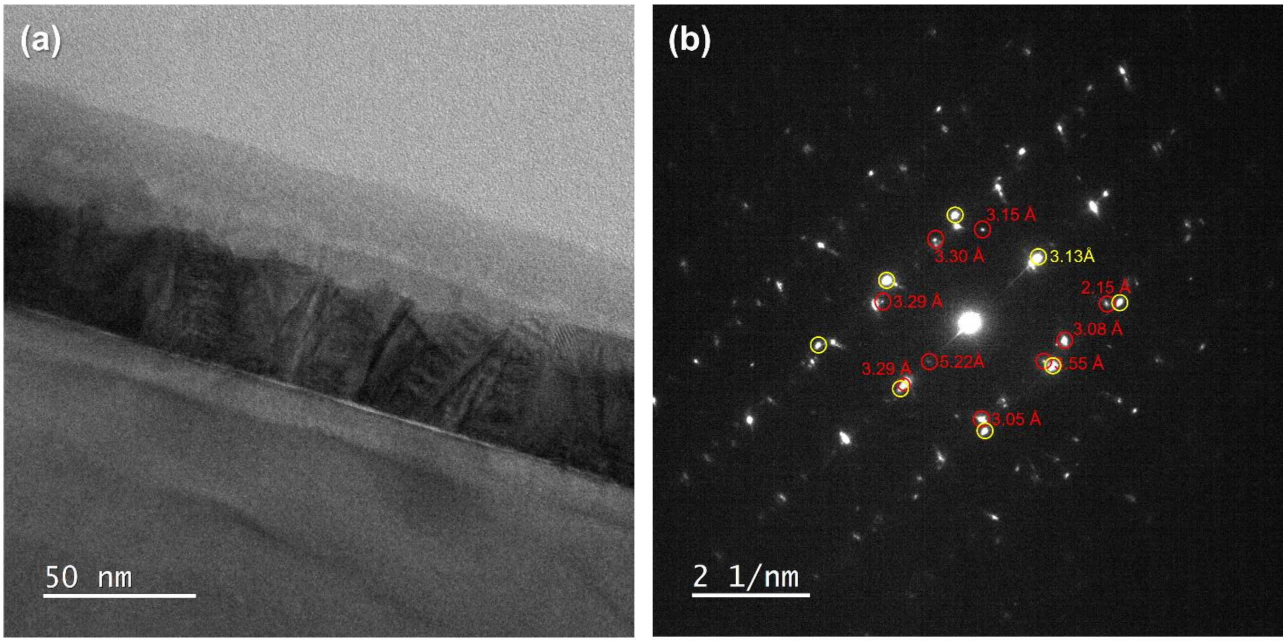

Figure S6. (**a**) Bright field TEM and (**b**) SAED of the sample H-Ge-GST on Si(111) after annealing at 330 °C. The SAED is obtained from a selected area of about 100 nm of diameter. Reflections marked in yellow correspond to the Si substrate along the [110] zone axis. The other reflections (in red) are compatible with Sb_2Te_3 and trigonal $(GeTe)_n(Sb_2Te_3)$.

References

1. Bruggeman, D.A.G. Berechnung verschiedener physikalischer Konstanten von heterogenen Substanzen. I. Dielektrizitätskonstanten und Leitfähigkeiten der Mischkörper aus isotropen Substanzen. *Ann. Phys.* **1935**, *416*, 636–664. (In German)
2. Kim, D.-H.; Merget, F.; Laurenzis, M.; Bolivar, P.H.; H. Kurz, H. Electrical percolation characteristics of $Ge_2Sb_2Te_5$ and Sn doped $Ge_2Sb_2Te_5$ thin films during the amorphous to crystalline phase transition *J. Appl. Phys.* **2005**, *97*, 083538.
